# Google Places API–Based US Built Environment Retail (UBER) Index and Its Spatial Association With Diabetes Prevalence Across US Counties: Cross-Sectional Ecological Study

**DOI:** 10.2196/95977

**Published:** 2026-10-07

**Authors:** Akshaya Srikanth Bhagavathula, Michelle A Williams

**Affiliations:** 1 Public Health Program, School of Health Systems & Innovation, College of Health and Human Sciences, North Dakota State University, NDSU Dept 2662, Fargo, ND, 58102, United States, 1 701-231-6549; 2 Department of Population Health and Epidemiology, School of Medicine, Stanford University, Stanford, CA, United States

**Keywords:** diabetes mellitus, built environment, digital epidemiology, spatial analysis, health disparities, geographic information systems, Google Places, Google

## Abstract

**Background:**

National surveillance of commercial retail environments remains limited by data sources that are updated infrequently and capture narrow dimensions of food access. The Google Places API provides continuously updated and programmatically accessible information on business locations across the United States, but its use as a population-level built-environment exposure measure has not been systematically evaluated.

**Objective:**

This study aims to develop and evaluate a Google Places–derived US Built Environment Retail (UBER) Index as a scalable measure of county-level commercial retail infrastructure in the United States and to estimate its spatial association with age-adjusted diabetes prevalence.

**Methods:**

We conducted a cross-sectional ecological study of contiguous US counties in accordance with the STROBE (Strengthening the Reporting of Observational Studies in Epidemiology) Statement. Counts of alcohol outlets, fast-food and convenience stores, grocery stores, and fitness and recreation facilities were extracted from the Google Places API in February 2026. Principal component analysis of 4 standardized indicators produced a composite index. Construct validity was assessed against benchmarks from the United States Department of Agriculture Food Access Research Atlas and County Health Rankings, with adequate convergence prespecified as |*r*|≥0.40. We estimated associations with age-adjusted diabetes prevalence from the Centers for Disease Control and Prevention PLACES 2025 dataset, using a spatial error model adjusted for the Area Deprivation Index, urbanicity, and census division. Quantile regression, county-versus-tract comparisons, and outcome-specificity analyses assessed robustness, outcome-resolution sensitivity, and specificity.

**Results:**

The analytic sample comprised 1701 of 2957 (57.5%) US counties. The first principal component explained 92.9% of variance, with near-equal loadings (0.492‐0.506). Convergent validity was weak (strongest Pearson *r*=–0.20; no comparison reached the prespecified threshold of absolute |*r*|≥0.40). Each 1-SD increase in the UBER Index was associated with 0.24 percentage points higher diabetes prevalence (95% CI 0.16‐0.32; *P*<.001), whereas area deprivation was the strongest predictor (0.086 per percentile; 95% CI 0.081‐0.091). Associations were stable across diabetes quantiles. The index was not associated with obesity and was inversely associated with coronary heart disease. The county-level association reversed at the tract level, indicating scale-dependent ecological confounding.

**Conclusions:**

Using programmatically accessible Google Places data, we developed the UBER Index as a scalable measure of county-level commercial retail infrastructure. This study is innovative because it shows how updated digital platform data can extend built-environment surveillance beyond static food-access measures. The index captures the broader commercial establishment volume and reveals spatial, distributional, and scale-dependent patterns relevant to diabetes research. It brings a new digital surveillance approach to built-environment epidemiology. In practice, it may help public health agencies monitor changing retail environments, while requiring longitudinal and individual-level validation before policy or practice use.

## Introduction

Diabetes affects more than 38 million adults in the United States and shows substantial geographic variation in prevalence that individual risk factors explain only in part [1]. A growing body of evidence links the built environment, meaning the physical and commercial structures in which populations live, work, and shop, to chronic disease risk through pathways involving food access, opportunities for physical activity, and exposure to health-relevant goods and services [2,3]. A recent systematic review and meta-analysis of longitudinal studies found that less healthful food environments were associated with higher incidence of type 2 diabetes, whereas greater walkability and green space were protective, underscoring the importance of where commercial and physical infrastructure is located in relation to diabetes burden [4]. This spatial patterning of commercial activity is increasingly framed within the “commercial determinants of health,” defined as the systems, practices, and pathways through which commercial actors influence health and equity [5]. Understanding how these characteristics vary across communities and relate to diabetes burden is essential for targeting prevention resources and designing place-based interventions [6,7]. Quantifying the geographic volume and concentration of retail commercial infrastructure is one way to operationalize this framework as a scalable exposure for population health research.

National-scale measurement of these commercial environments, however, remains constrained by data limitations. Widely used sources such as the United States Department of Agriculture (USDA) Food Access Research Atlas [8], the County Health Rankings food environment index [9], and the Modified Retail Food Environment Index were designed primarily to characterize food access and food-insecurity deprivation, capture narrow dimensions of the food environment, are updated infrequently, and rely on administrative records that may lag behind actual commercial conditions. It is important to distinguish 2 related but nonidentical constructs at the outset: the food environment, meaning the availability and accessibility of food outlets relevant to diet, and the broader overall commercial establishment volume and concentration of a community, meaning the intensity and diversity of retail activity across sectors. Existing tools were designed to characterize the former; no national surveillance system provides a comprehensive, frequently updated measure of the latter across US counties.

Digital platform data may offer a potential solution to this measurement gap [10]. Commercial mapping services, particularly the Google Places API, maintain continuously updated databases of business locations and attributes across the United States. These data are programmatically accessible, geographically referenced, and available at fine spatial resolution. Several studies have used Google-derived data for local built-environment assessments [11,12], and ecological analyses continue to link commercial food-outlet density to diabetes burden [13], but no study has systematically evaluated whether API-derived retail measures can function as valid, epidemiologic exposures at the national level. Addressing this gap is primarily a methodological problem: whether continuously updated digital platform data can be transformed into a standardized, spatially structured exposure suitable for national surveillance.

Accordingly, the aim of this study was to develop and evaluate a Google Places−derived US Built Environment Retail (UBER) Index as a scalable measure of county-level commercial retail infrastructure in the contiguous United States, and to characterize its spatial relationship with age-adjusted diabetes prevalence. We pursued four objectives: (1) to construct the UBER Index from Google Places establishment counts and characterize its internal structure, (2) to evaluate its construct validity against established food-environment benchmarks, (3) to estimate its spatial association with county-level age-adjusted diabetes prevalence using spatial models that account for geographic dependence and area-level confounders, and (4) to test the robustness of that association across the outcome distribution and across geographic scale. Given the cross-sectional, ecological design, we interpreted the findings cautiously. We hypothesized that the index would (1) capture a general dimension of commercial retail volume distinct from existing food-access measures and (2) show a measurable but scale-dependent association with diabetes prevalence. Figure 1 presents the conceptual framework for the UBER study, illustrating the data-to-evidence pipeline from Google Places API inputs through spatial modeling to population inference, including the structural boundaries imposed by the ecological design.

**Figure 1.**
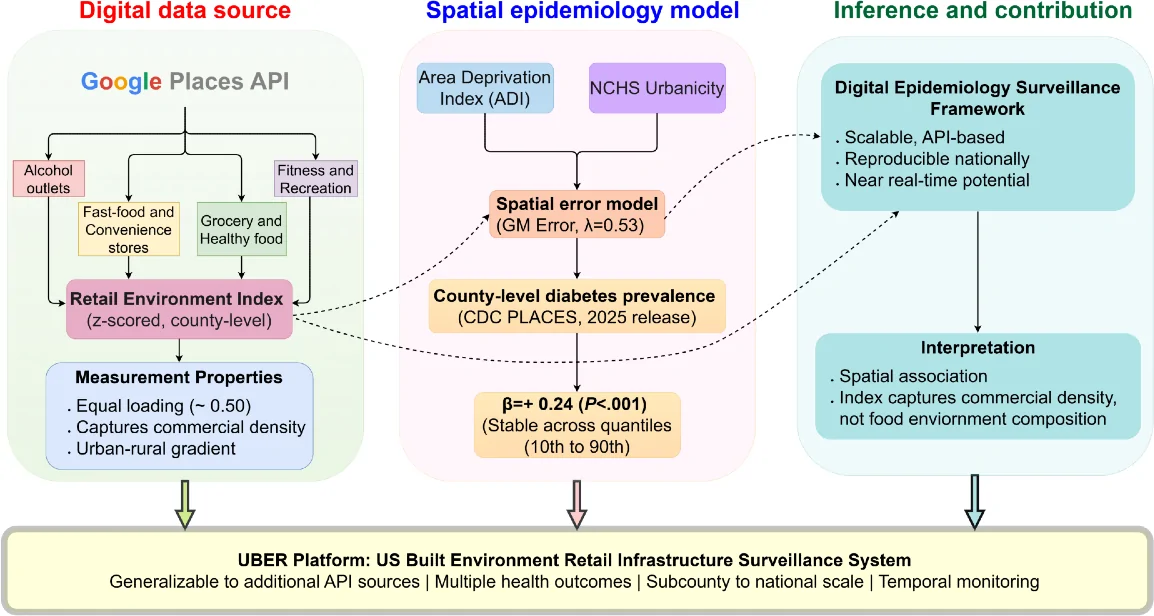
Conceptual framework for the study, showing the data-to-evidence pipeline for the US Built Environment Retail (UBER) Index. The schematic traces the flow from Google Places API inputs (February 2026), through the construction of a principal-component exposure index and spatial modeling, to population-level inference for age-adjusted diabetes prevalence across counties of the contiguous United States and highlights the structural boundaries imposed by the cross-sectional ecological design. CDC: Centers for Disease Control and Prevention; NCHS: National Center for Health Statistics.

## Methods

### Study Design

We conducted a cross-sectional ecological study examining county-level commercial retail infrastructure and diabetes prevalence across the contiguous United States, and we report it in accordance with the STROBE (Strengthening the Reporting of Observational Studies in Epidemiology) guideline for cross-sectional studies (completed checklist provided as Checklist 1).

### Setting

The unit of analysis was the county (or county-equivalent). The setting comprised all counties of the contiguous United States; establishment data were extracted from the Google Places API in February 2026 and were linked to age-adjusted diabetes prevalence from Centers for Disease Control and Prevention (CDC) PLACES (2025 release) [14], the Area Deprivation Index (ADI) national percentile ranking (2023) [15], and the National Center for Health Statistics (NCHS) urban-rural classification [16].

### Participants

The study sought complete enumeration of all US counties rather than drawing a probability sample. Counties in Alaska, Hawaii, and US territories were excluded a priori because contiguity-based spatial weights require geographic adjacency within a single connected landmass. Because the analysis required complete data across all exposure, outcome, and covariate variables, we used a complete-case approach; counties missing one or more Google Places establishment categories or any covariate were excluded. The final analytic sample comprised 1701 counties with complete data, approximately 57.5% of the 2957 contiguous-US counties with CDC PLACES estimates (and 54.1% of all 3143 US counties and county-equivalents). Because missingness in Google Places coverage was associated with rurality and population, we did not impute excluded counties; instead, we quantified the proportion excluded for each reason and compared included vs excluded counties on census region, urbanicity, area deprivation, and diabetes prevalence to assess potential selection bias.

### Variables

#### Exposure Variable

The primary exposure was the UBER Index, a county-level composite measure of commercial retail infrastructure derived from Google Places API data.

#### Outcome

The primary outcome was county-level age-adjusted diabetes prevalence from CDC PLACES (2025 release).

#### Covariates

Models were adjusted for 3 categories of contextual factors. Socioeconomic deprivation was measured using the ADI national percentile ranking (range 1 to 100). Urbanicity was included as a set of indicator variables for NCHS urban-rural classification codes (6 categories; large central metro as reference). Regional variation was addressed with Census division fixed effects (9 divisions; New England as reference).

#### Variables Used for Construct Validation

To evaluate construct validity, the UBER Index was compared with established food-environment benchmarks, including the following:

- USDA Food Access Research Atlas indicators
- County Health Rankings food insecurity estimates
- Supplemental Nutrition Assistance Program (SNAP) retailer density

### Data Sources and Measurement

#### Exposure Measurement

We obtained commercial retail establishment data using the Google Places API Nearby Search end point in February 2026. Nearby search requests were centered on county centroids using a fixed 3-km radius catchment (approximately 28.3 km^2^) that was applied uniformly across all counties. This design represents a fixed-radius centroid catchment rather than a county-wide census of establishments. Consequently, in geographically large counties, the catchment samples the central, typically more populous and commercially active, portion of the county rather than its entire area, whereas in counties smaller than the catchment, it may include establishments located just beyond the county boundary. Because the identical protocol was applied to every county, any resulting spatial misclassification is expected to be nondifferential with respect to diabetes prevalence and would therefore bias associations toward the null. The fixed-radius approach also implicitly standardizes the queried area across counties, providing a measure of establishment concentration within a constant catchment area.

For each county centroid, we queried 4 categories of establishments relevant to health behaviors: alcohol-serving establishments, fast food and convenience stores, grocery and health food stores, and fitness and recreation facilities. The 4 domains were defined using prespecified Google Places types. Alcohol outlets included liquor_store, bar, night_club, pub, and beer_garden. Fast-food and convenience outlets included fast_food_restaurant, convenience_store, meal_takeaway, and meal_delivery. Grocery and health-food outlets included supermarket, grocery_or_supermarket, grocery_store, and food with grocery keyword constraints. Fitness and recreation outlets included gym, fitness_center, sports_complex, park, community_center, bowling_alley, and swimming_pool. For each coordinate, the first page of results was retrieved, with up to 20 results per query; next_page_token was not used. Duplicate establishments were removed using unique place_id values within each category before aggregation. Establishments tagged in more than one category contributed to each relevant subdomain count, while the composite index used place_id deduplication across categories to avoid double-counting. Returned points were assigned to the centroid catchment rather than clipped to county polygons. Thus, the measure represents commercial establishment volume within a fixed county-centroid catchment rather than a whole-county count.

We constructed the UBER Index from these establishment counts to represent overall commercial retail infrastructure at the county level. These 4 categories were selected a priori to span the principal retail domains theorized to shape diet- and activity-related behaviors relevant to diabetes: outlets associated with health-adverse consumption, outlets providing access to foods for home preparation, and outlets supporting physical activity. Together, they were intended to capture the intensity and mix of health-relevant commercial retail activity in a county rather than any single food-access dimension.

Raw counts were winsorized at the 1st and 99th percentiles, log-transformed, and standardized as *z* scores. We applied principal component analysis (PCA) to the 4 standardized indicators and retained the first principal component as the UBER Index. The index was standardized to mean 0 and SD 1. We evaluated internal consistency using Cronbach α, computed for the 4-indicator set and separately within the health-adverse (alcohol outlets, fast-food, and convenience stores) and health-promoting (grocery and health-food stores, fitness, and recreation facilities) subdomains.

#### Outcome Measurement

The primary outcome was county-level age-adjusted diabetes prevalence obtained from the CDC PLACES 2025 release. CDC PLACES provides model-based estimates derived from Behavioral Risk Factor Surveillance System (BRFSS) data for all US counties. Diabetes prevalence was analyzed as a continuous variable representing the percentage of adults aged 18 years or older with diagnosed diabetes.

#### Covariate Measurement

Socioeconomic deprivation was measured using the ADI national percentile ranking (2023). Urbanicity was measured using the NCHS urban-rural classification scheme for counties. Regional variation was captured using Census division classifications and included as fixed effects in regression models.

#### Construct-Validation Measures

Construct validity of the UBER Index was evaluated against established food-environment benchmarks, including indicators from the USDA Food Access Research Atlas [17], County Health Rankings food insecurity estimates [18], and SNAP retailer density [19]. Correlations between the UBER Index and these benchmark measures were examined to assess convergent validity.

### Bias

Several measures were taken to address potential sources of bias. Standardized national datasets and prespecified analytic procedures were used for exposure construction and model selection. To evaluate potential selection bias arising from incomplete Google Places coverage, included and excluded counties were compared on region, urbanicity, area deprivation, and diabetes prevalence. Spatial error models (SEMs) were used to account for spatial dependence, and sensitivity analyses examined robustness across geographic scales and outcome distributions. Nevertheless, residual confounding, ecological bias, exposure misclassification, and selection bias related to incomplete data coverage remain possible.

### Study Size

The study sought complete enumeration of all eligible contiguous US counties rather than sampling. Therefore, no formal sample-size calculation was performed. All counties meeting eligibility and data-completeness criteria were included in the analysis.

### Quantitative Variables

Establishment counts were winsorized at the 1st and 99th percentiles, log-transformed, and standardized as *z* scores before PCA. The resulting UBER Index was standardized to a mean of 0 and SD of 1. Diabetes prevalence and ADI percentile rankings were analyzed as continuous variables.

### Statistical Methods

#### Missing Data

We assessed whether missing Google Places exposure data were missing completely at random (MCAR) using Little’s MCAR test. We also modeled the probability of missing exposure using county rurality, population, and diabetes prevalence. Because missing exposure occurred at the county level and counties without exposure data had no within-unit exposure information to impute, complete-case analysis was used. Included and excluded counties were compared to assess potential selection bias.

#### Spatial Modeling

We first assessed spatial autocorrelation using Global Moran *I* on model residuals and applied the Anselin decision rule based on Lagrange multiplier (LM) specification tests to select between spatial lag and spatial error specifications [20]. LM tests indicated spatial error dependence for diabetes (LM-error=640.2, *P*<.001; robust LM-error=652.7, *P*<.001), supporting an SEM specification [21].

The primary model was an SEM estimated via generalized method of moments (GM-Error), which accounts for spatially structured residual dependence through a spatial autoregressive error parameter (*λ*). Neighborhood structure was defined using first-order queen contiguity weights, which were row-standardized. Among the 1701 analytic counties, 27 had no contiguous neighbor, and the weight graph included 51 connected components. Island counties were retained in the model with a zero spatial lag. To assess robustness, we repeated the SEM using k-nearest-neighbor weights (*k*=6) and 100-km distance-band weights. An ordinary least squares (OLS) baseline was estimated for comparison. We report regression coefficients, standard errors, *z*-statistics, and pseudo-*R*^2^ for the SEM alongside the OLS *R*^2^.

#### Sensitivity Analyses

We conducted 4 sensitivity analyses. First, we estimated quantile regression models at the 10th, 25th, 50th, 75th, and 90th percentiles to examine variation across the distribution of diabetes prevalence. Second, we conducted an outcome-resolution sensitivity analysis by comparing county-level OLS estimates with tract-level random-intercept multilevel models (tracts nested within counties) using the same county-level UBER Index value assigned to each constituent tract, adjusting for ADI. Because exposure values were held constant within counties, this analysis was intended to evaluate sensitivity to outcome resolution and model specification rather than within-county exposure variation. Third, as an exploratory outcome-specificity analysis, we reestimated the primary SEM with age-adjusted obesity and coronary heart disease prevalence (CDC PLACES, 2025) as alternative outcomes, using the same exposure and covariate specification. Fourth, to evaluate the extent to which the UBER Index reflected population or market size, we reestimated the primary SEM after adding log-transformed county population as an additional covariate.

#### Software

All analyses were conducted in Python 3.11 using PySAL/spreg for spatial modeling, scikit-learn for PCA, statsmodels for regression models, and geopandas for spatial data management. All statistical tests were 2-sided, with statistical significance set at *α*=.05.

### Ethical Considerations

This study analyzed only publicly available, deidentified, aggregate data at the county and census-tract level and therefore did not constitute human participants research; it was exempt from institutional review board review, and no approval or waiver was required. No individuals were enrolled, contacted, or intervened upon, so informed consent and participant compensation were not applicable. The secondary sources used, including CDC PLACES (derived from the BRFSS), were collected under their own consent and governance frameworks and released publicly in deidentified, aggregate form, which permits secondary analysis without additional consent. No personally identifiable information was accessed or generated at any stage, and no individuals are identifiable in any figure.

## Results

### Study Sample

The analytic sample comprised 1701 counties in the contiguous United States with complete exposure, outcome, and covariate data, representing 57.5% of the 2957 contiguous US counties for which CDC PLACES estimates were available (and 54.1% of all 3143 US counties and county-equivalents). The 1256 excluded contiguous US counties lacked complete data for 1 or more variables, most commonly incomplete Google Places coverage for at least 1 establishment category (42.5% of 2957 contiguous-US counties) or the ADI. To assess potential selection bias, we compared included with excluded counties. Excluded counties were substantially more rural (85.0%, 1068/1256 vs 45.4%, 772/1701 in NCHS categories 5-6), somewhat more concentrated in the Midwest (41.7%, 524/1256 vs 31.2%, 531/1701), and had marginally higher age-adjusted diabetes prevalence (mean 11.4%, SD 2.4% vs 11.0%, SD 2.2%; *P*<.001; Multimedia Appendix 1). The analytic sample therefore included a larger share of urban and populous counties.

Missing data were limited to the Google Places exposure, which was incomplete for 1256 of 2957 (42.5%) contiguous United States counties. Little’s MCAR test rejected the MCAR null hypothesis (*P*<.001). In logistic regression, missing exposure was more likely in rural counties (OR 2.73, 95% CI 2.2‐3.4; *P*<.001) and less likely in counties with larger populations (OR 0.52 per log-unit increase; *P*<.001), but it was not independently associated with diabetes prevalence (*P*=.29). These findings indicate that missingness was not MCAR and was primarily related to observable geographic characteristics.

### Index Measure Properties

The final principal component explained 92.9% of total variance across the 4 Google Places indicators, capturing nearly all covariation in establishment counts within a single dimension of general commercial volume a concentration; this variance refers to the exposure measurement space rather than diabetes prevalence. Loadings were similar across indicators: grocery and health food stores (0.506), fast food and convenience stores (0.503), alcohol outlets (0.499), and fitness and recreation facilities (0.492; Figure 2A). The structure indicates that the index reflects overall commercial retail volume rather than distinguishing between health-promotion and health-adverse retail types.

**Figure 2.**
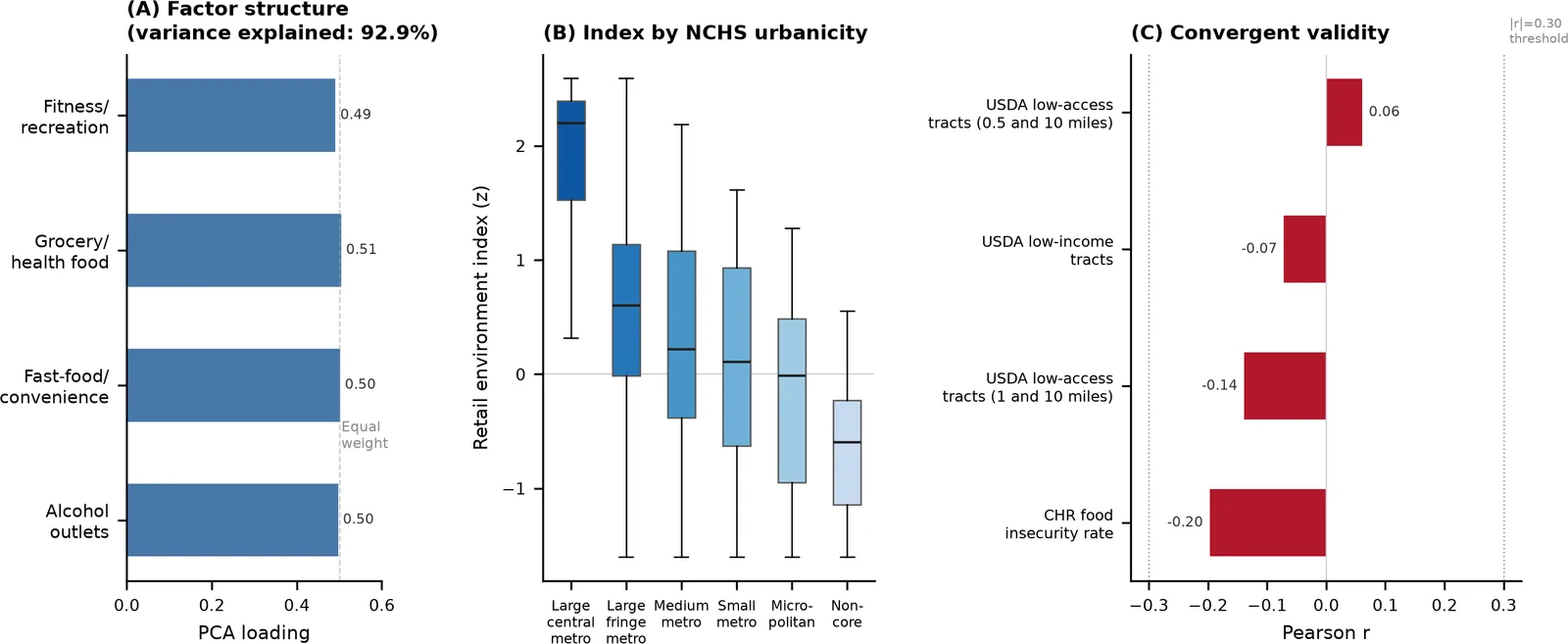
Construct validity and internal structure of the US Built Environment Retail (UBER) Index across 1701 counties of the contiguous United States (cross-sectional ecological study; Google Places API, February 2026). Panels show (A) principal-component loadings of the 4 retail indicators, (B) the distribution of the index across National Center for Health Statistics (NCHS) urban-rural categories, and (C) correlations between the index and established food-environment benchmarks (United States Department of Agriculture [USDA] Food Access Research Atlas; County Health Rankings [CHR]), none of which reached the prespecified convergent-validity threshold (absolute *r* of at least 0.40). PCA: principal component analysis.

The index exhibited a clear urban-rural gradient. Large central metro counties (NCHS code 1) had the highest index values, and values declined toward noncore rural counties (NCHS code 6; Figure 2B). Internal consistency was high (Cronbach *α*=.95) for the full 4-indicator set. Cronbach α was .95 for the health-adverse retail subdomain and 0.94 for the health-promoting retail subdomain.

Convergent validity with established food environment benchmarks was weak. The UBER Index correlated most strongly with the County Health Rankings food insecurity rate (Pearson *r*=−0.20; *P*<.001; n=1701); counties with a denser commercial establishment volume and concentration tended to have somewhat lower food insecurity, consistent with the concentration of both retail activity and economic resources in urban areas. Correlations with USDA Food Access Research Atlas indicators were weaker (|*r*|=0.06-0.14; Figure 2C). No benchmark comparisons reached the prespecified threshold of |*r*|≥0.40, used to indicate adequate convergent validity. These results indicate that the UBER Index does not measure food access or food insecurity as traditionally defined but instead captures a distinct dimension of the commercial built environment: the overall intensity of retail activity in a county.

### Spatial Distribution

The UBER Index showed substantial spatial clustering across the contiguous United States (Figures 3 and 4). Global Moran *I* for the index was 0.44 (*P*=.001), indicating significant positive spatial autocorrelation. Higher index values occurred primarily in metropolitan regions along the eastern seaboard, Great Lakes, and Pacific coast, whereas lower values were observed in rural regions of the Great Plains, Appalachian interior, and Mountain West. This regional patterning indicates that the commercial retail environments captured by the index vary systematically across broad geographic areas, with implications for the spatial distribution of any associated health outcomes.

**Figure 3.**
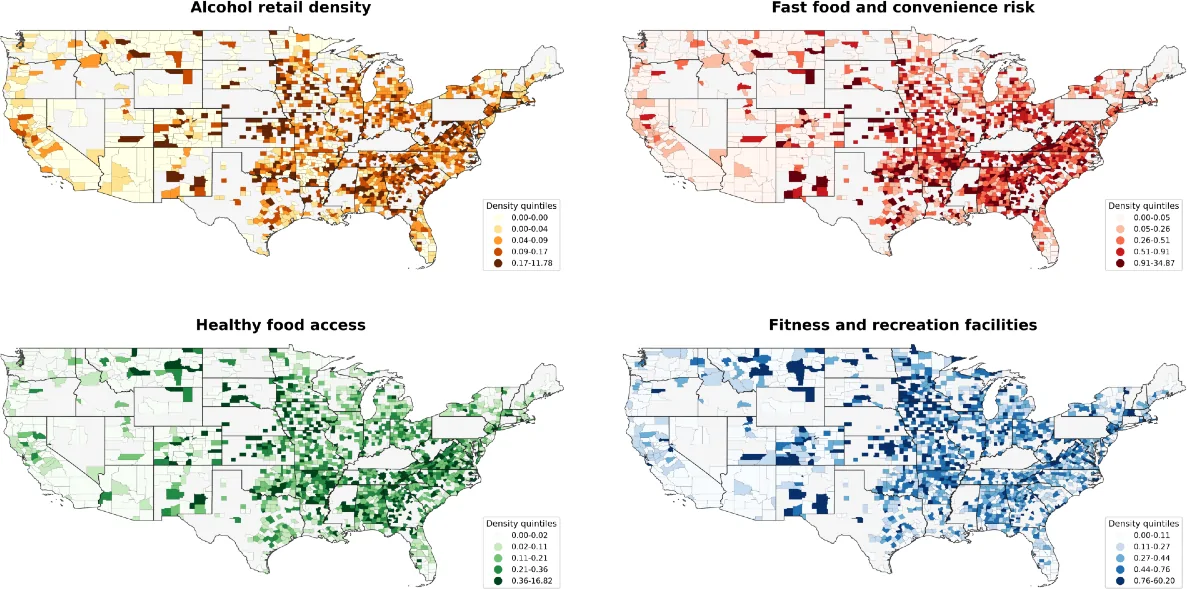
Spatial distribution of the US Built Environment Retail (UBER) Index (density per 1000 residents) across 1701 counties of the contiguous United States (cross-sectional ecological study; Google Places API, February 2026). Maps display the geographic patterning and clustering of commercial retail volume used in the subsequent spatial models of age-adjusted diabetes prevalence. County-level distribution of the 4 Google Places-derived retail indicators (alcohol outlets; fast-food and convenience stores; grocery and health-food stores; fitness and recreation facilities) across the contiguous United States (1701 counties; February 2026).

**Figure 4.**
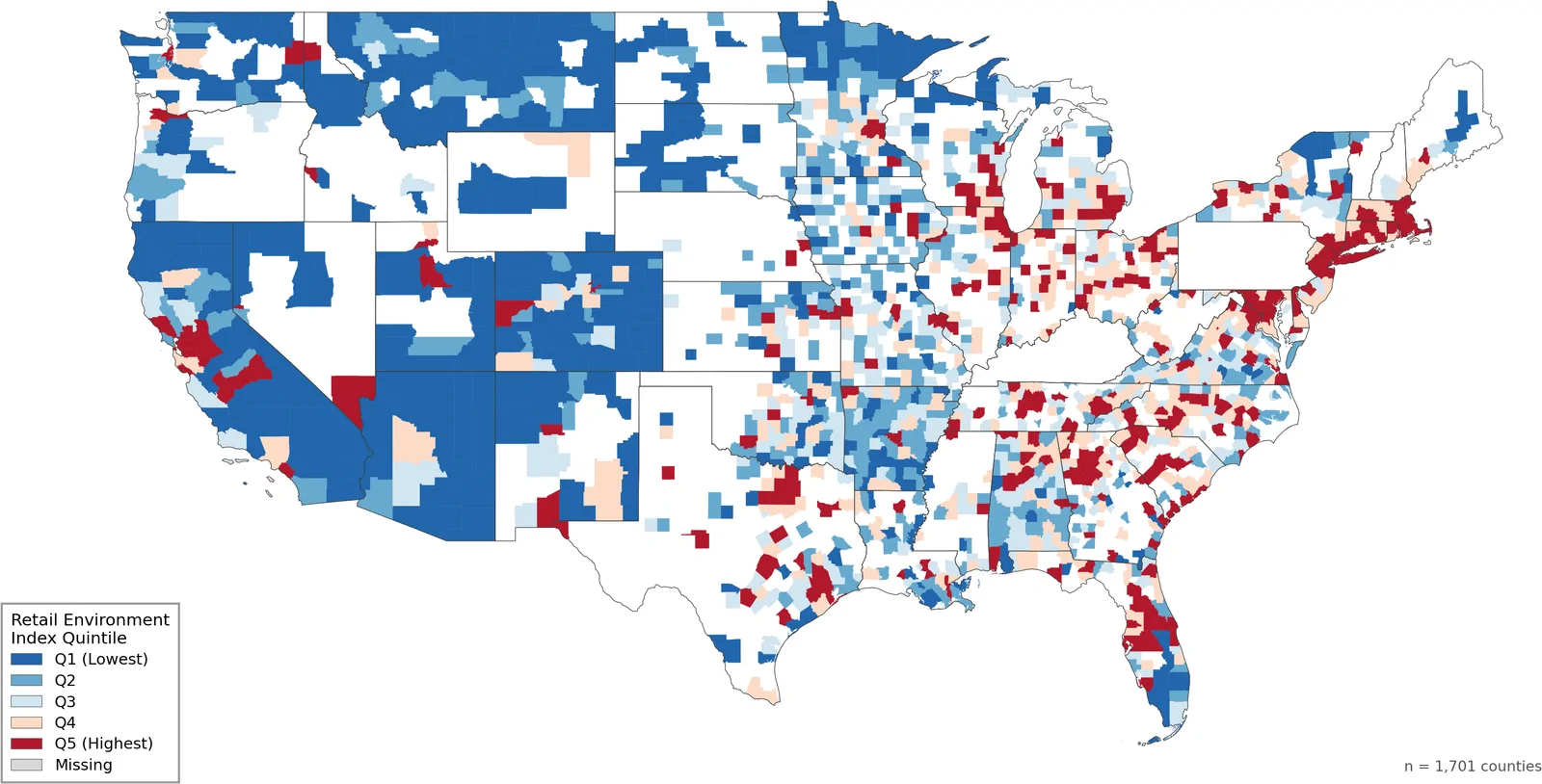
County-level choropleth of the US Built Environment Retail (UBER) Index (quintiles) across 1701 counties of the contiguous United States, February 2026.

### SEM: Diabetes Association

Diabetes prevalence also showed strong spatial autocorrelation (Moran *I*=0.59; *P*=.001); counties with high prevalence tended to neighbor other high-prevalence counties. Lagrange multiplier tests supported a spatial error specification. In that model, each 1-SD increase in the UBER Index was associated with a 0.24 percentage-point higher county-level diabetes prevalence (β=.240; SE=0.041; *z*=5.88; 95% CI 0.16-0.32; *P*<.001) after adjustment for the ADI, NCHS urbanicity, and census division (Figure 5). The spatial autoregressive error parameter (*λ*) was 0.534, indicating substantial spatially structured residual dependence. The SEM pseudo-*R*^2^ was 0.572, and the OLS *R*^2^ was 0.582; these values are not directly comparable because pseudo-*R*^2^ in SEM partitions variance differently than OLS *R*^2^, and a lower value does not indicate worse model fit. In parallel SEMs using the same index and covariates, the UBER Index was not associated with age-adjusted obesity prevalence (β=−.06; 95% CI −0.24 to 0.13; *P*=.55) and was at most weakly and inversely related to coronary heart disease prevalence (β=−0.03; 95% CI −0.05 to 0.001; *P*=.06). This outcome specificity is consistent with the diabetes association not being a generic correlate of urbanization shared uniformly across chronic conditions, although we interpret this exploratory comparison cautiously.

**Figure 5.**
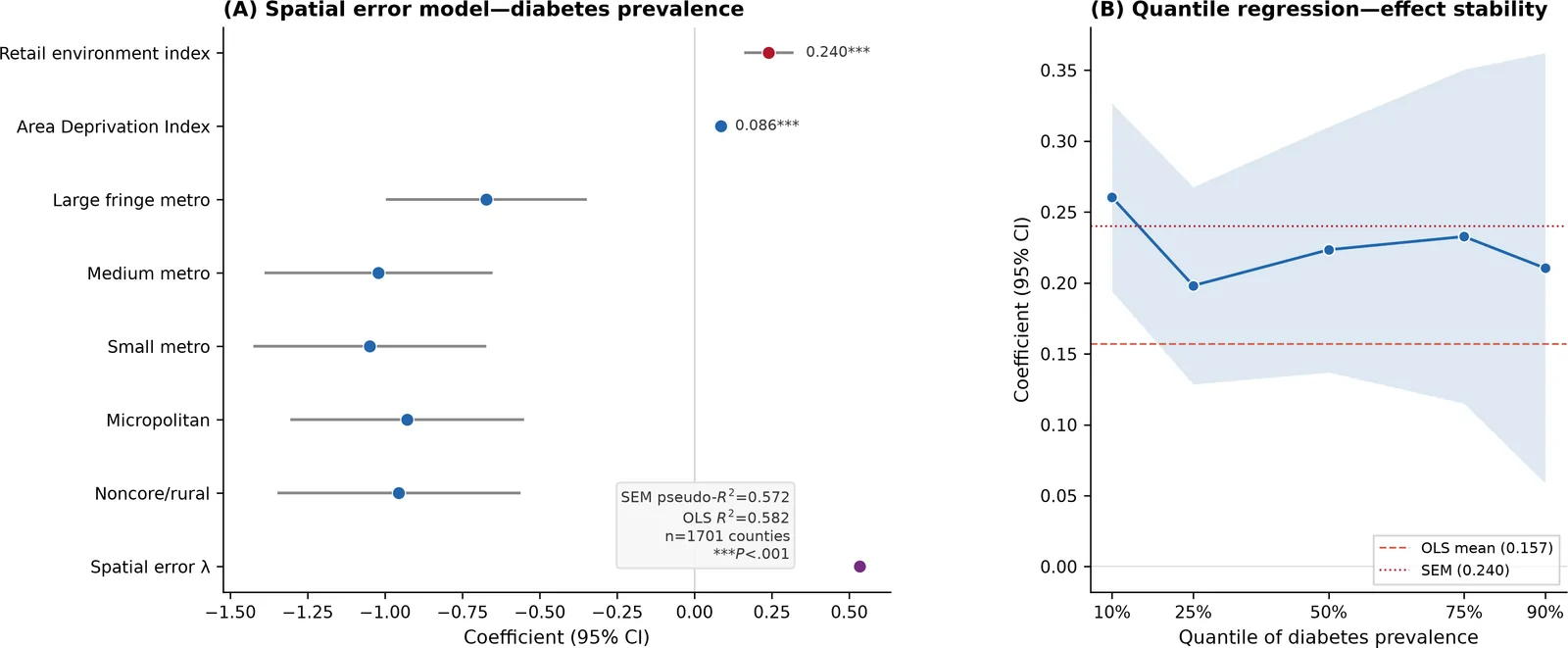
Association between the US Built Environment Retail (UBER) Index and county-level age-adjusted diabetes prevalence (Centers for Disease Control and Prevention [CDC] PLACES, 2025) across 1701 counties of the contiguous United States (cross-sectional ecological study). (A) SEM estimates (generalized method of moments; queen contiguity weights) adjusted for the Area Deprivation Index, urbanicity, and census division, with 95% CIs. (B) Quantile-regression coefficients for the index across the 10th to 90th percentiles of diabetes prevalence, with 95% CIs. OLS: ordinary least squares; SEM: spatial error model.

After adjusting for log-transformed county population, the association between the UBER Index and diabetes prevalence was reduced yet remained statistically significant (*β*=.121; 95% CI 0.03‐0.21; *P*=.007). County population showed an independent positive association with diabetes prevalence (*β*=.220; *P*<.001).

The association was robust to alternative spatial-weight definitions. The UBER coefficient was similar using queen contiguity weights (*β*=.240; *P*<.001), k-nearest-neighbor weights (*k*=6; *β*=.238; *P*<.001), and 100-km distance-band weights (*β*=.195; *P*<.001). Global Moran *I* for diabetes was also similar across weight schemes (0.593, 0.591, and 0.595, respectively), indicating that the association did not depend on the selected contiguity definition.

Area deprivation was the strongest predictor. Each 1-unit increase in ADI national percentile was associated with a 0.086 percentage-point increase in diabetes prevalence (95% CI 0.081-0.091; *P*<.001). Compared with large central metro counties, all nonmetro urbanicity categories were associated with lower diabetes prevalence after adjustment for ADI and the retail index, ranging from −0.67 (95% CI −1.00 to −0.35) to −1.05 (95% CI −1.43 to −0.67) percentage points (all *P*<.001).

### Quantile Regression

The association between the UBER Index and diabetes prevalence remained stable across the conditional distribution of diabetes (Figure 5). Coefficients were 0.260 (95% CI 0.194-0.327) at the 10th percentile, 0.198 (95% CI 0.129-0.268) at the 25th, 0.223 (95% CI 0.137-0.310) at the 50th, 0.233 (95% CI 0.115-0.350) at the 75th, and 0.210 (95% CI 0.059-0.362) at the 90th (all *P*≤.007). CIs overlapped across all quantiles, indicating the association was not driven by counties with the highest or lowest diabetes prevalence.

### Outcome-Resolution Sensitivity Analysis

Outcome-resolution sensitivity analysis revealed discordant results across geographic levels (Figure 6). At the county level, the UBER Index was positively associated with diabetes (*β*=.353; *P*<.001; n=1701 counties). At the tract level, the index was negatively associated with diabetes in random intercept models using the county-level UBER Index assigned to all tracts within each county (β=−.281; *P*=.034; n=17,581 tracts). Because the county-level UBER Index was assigned unchanged to all tracts within a county, this analysis introduced no within-county exposure variation. Therefore, the observed sign reversal should not be interpreted as evidence of a within-county relationship or a cross-level effect, but rather as evidence that the association was sensitive to outcome resolution and model specification.

**Figure 6.**
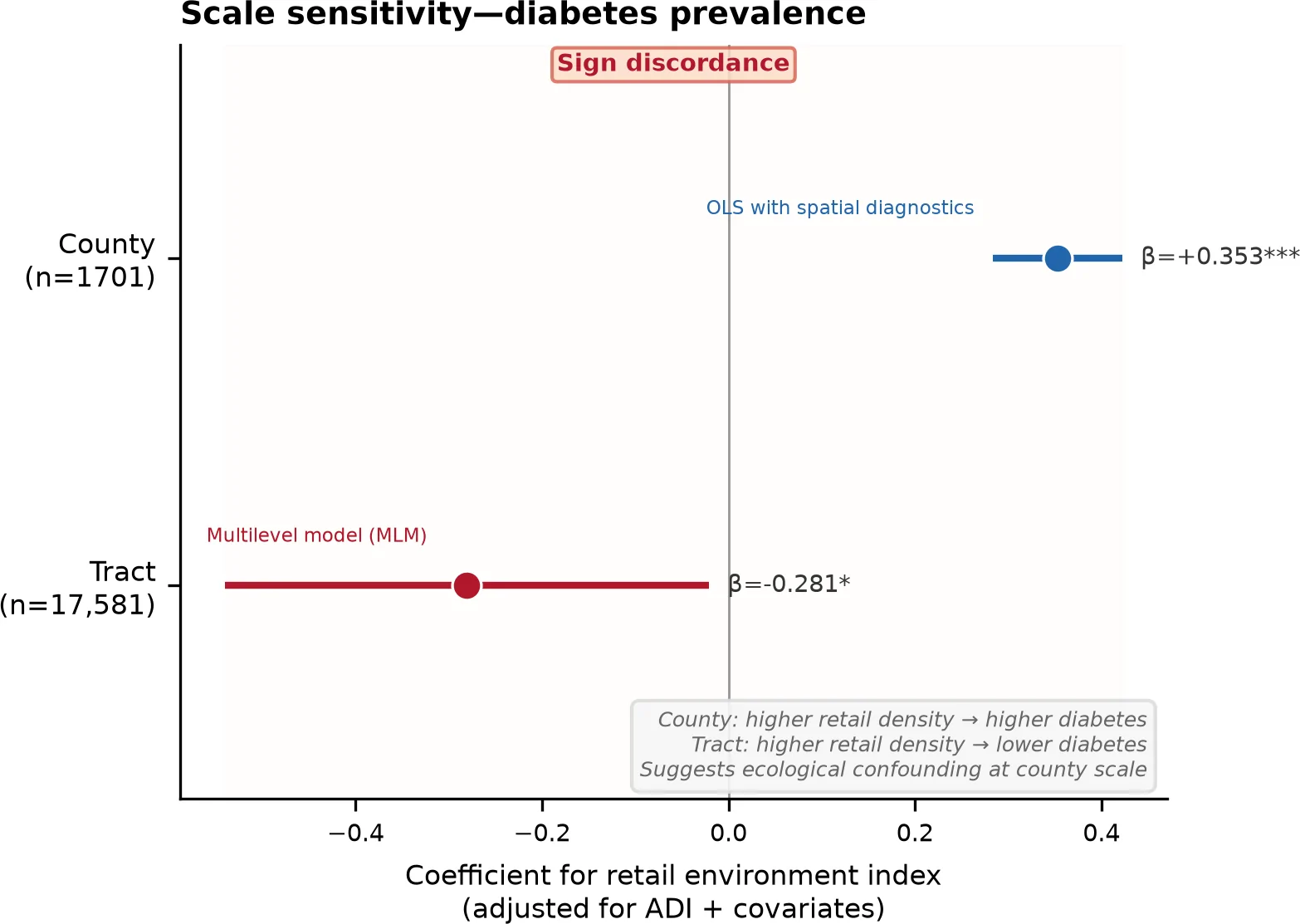
Outcome-resolution sensitivity analysis comparing county-level (n=1701) and tract-level models (n=17,581). Because the county-level US Built Environment Retail (UBER) Index was assigned unchanged to all tracts within a county, the analysis evaluates sensitivity to outcome resolution and model specification rather than within-county exposure variation. ADI: Area Deprivation Index; OLS: ordinary least squares. *P=.03, ***P<.001.

## Discussion

### Principal Findings

In this national ecological study of counties in the contiguous United States, we set out to construct a Google Places−derived index of commercial retail infrastructure, evaluate its measurement properties, and characterize its spatial relationship with age-adjusted diabetes prevalence. The UBER Index behaved as a coherent, spatially structured measure and showed a positive county-level association with diabetes prevalence that was stable across the distribution of diabetes and consistent in both OLS and spatial error specifications. At the same time, the analysis surfaced 2 findings that constrain interpretation: the index aligned only weakly with established food-environment benchmarks, and its association with diabetes reversed direction between the county and tract scales. Read together, these results position the study primarily as a methodological demonstration that programmatically accessible platform data can be turned into an exposure surface for county-level population health research, pending external validation, rather than as evidence of an etiologic link between commercial establishment volume and concentration and diabetes.

The PCA loading structure provided clear evidence about what the index captured. All 4 Google Places−derived indicators loaded near equally on a single dominant component, which explained 92.9% of total covariation among the 4 exposure indicators. This pattern indicated that the UBER Index represented general commercial establishment volume and concentration rather than a specific food-environment construct. Similar PCA applications have shown that multiple outlet indicators often collapse into shared dimensions that reflect broader neighborhood retail structure rather than specific food categories [22]. Counties with higher scores had more establishments across all categories, likely reflecting the spatial colocation of retail outlet types and broader commercial development. Retail outlet types frequently co-locate spatially, producing correlated establishment counts across categories such as alcohol, tobacco, and other commercial establishments [23], which can make composite indices reflect overall commercial development rather than specific food-access conditions [24]. The attenuation after adjustment for county population further supported this interpretation. Because the UBER Index was constructed from establishment counts rather than population-standardized measures, it partly reflected population and market size. However, the association remained statistically significant after population adjustment, suggesting that the index captured variation beyond population size alone.

The monotonic urban-rural gradient observed in Figure 2B reinforces this interpretation. Large central metro counties scored the highest; noncore rural counties scored the lowest. Research on food retail accessibility shows that urban areas typically have substantially higher counts and diversity of food outlets than rural areas [25]. The UBER Index therefore appears to reflect commercial establishment volume and concentration, which is partly related to population and market size but not fully explained by it. These characteristics differ fundamentally from food-access deprivation (USDA) or food insecurity (Feeding America). The weak convergent validity confirms this distinction empirically, consistent with methodological reviews showing that neighborhood food-environment indicators often reflect structural retail availability rather than direct measures of food insecurity [24]. Because excluded counties were disproportionately rural, these findings may not generalize to all counties in the contiguous United States.

The county-level association between the UBER Index and diabetes prevalence was statistically robust and directionally consistent between OLS and spatial error specifications, though modest in population terms. Ecological studies using county-level data have similarly reported associations between retail food-outlet availability and diabetes prevalence across US counties [26], and the broader literature links less healthful food environments to higher type 2 diabetes incidence [4]. The substantial spatial error parameter indicates that residual spatial dependence, likely reflecting unmeasured spatially structured confounders such as area-level socioeconomic composition and behavior, is an important feature of the data; spatial clustering of diabetes and its socioecological correlates is well documented in geographic analyses of US counties [27]. Because the design is ecological, this county-level association is best read as a population-level correlation rather than an individual-level or causal effect, and most plausibly reflects the colocation of commercial establishment volume and concentration with urbanization and deprivation rather than a direct effect of retail exposure.

The quantile regression analysis provides evidence against a common concern in ecological studies that observed associations are driven by extreme values [28]. The coefficient for the UBER Index was remarkably stable across the 10th through 90th percentiles of diabetes prevalence, with broadly overlapping CIs, suggesting a consistent population-level spatial pattern rather than an artifact of outlier counties. This stability is consistent with reviews showing that food-environment associations are not confined to extreme contexts [29].

When the county-level UBER Index was applied unchanged to census tracts and compared with tract-level diabetes prevalence, the coefficient reversed sign. Because this analysis held exposure constant within each county, it introduced no within-county exposure variation and therefore cannot demonstrate a within-county relationship, a cross-level effect, or a formal modifiable areal unit problem [30]. The observed sign change may instead reflect differences in outcome resolution, tract-level weighting, and model specification. We therefore restrict interpretation to the county scale and treat this finding only as evidence that the observed association is sensitive to analytic resolution. Establishing genuine multiscale relationships will require tract-level exposure measures derived directly from establishment data, which we identify as an important priority for future research [29,30].

The primary contribution of this study is methodological rather than etiologic. We showed that Google Places API data can be systematically transformed into a standardized, scalable built-environment exposure index, with clear internal structure, high internal consistency, and significant spatial autocorrelation. Recent methodological reviews note that emerging digital spatial datasets and computational tools are increasingly used to measure neighborhood environments and generate large-scale exposure variables [31-33]. These findings provide a proof of concept for the UBER framework: an API-based system for monitoring commercial retail environments at the county level; more broadly, API-accessible platform data are increasingly proposed for broader monitoring of commercial exposure environments [34].

The UBER framework has 3 operational advantages over traditional built-environment data systems. First, Google Places data are programmatically queryable, enabling standardized exposure construction across thousands of geographic units without manual data collection. Second, the underlying data source is updated continuously as businesses open, close, or change type, creating the potential for near-real-time environmental monitoring, a capability absent from existing food-environment datasets that are updated at multiyear intervals [35]. Third, the framework is generalizable: the same API infrastructure can support queries for additional establishment types, finer geographic resolutions, and temporal trend analyses. Points-of-interest datasets are increasingly used as scalable digital representations of urban commercial activity for public health and urban research [36].

The weak convergent validity with USDA and food-insecurity benchmarks admits more than 1 interpretation, and we read it cautiously. On one reading, the index captures a dimension of the built environment, general commercial retail volume, that existing food-access surveillance tools do not represent. On another, equally consistent reading, the weak correlations simply indicate that the index aligns only loosely with the food-environment constructs it was intended to approximate and that it may index overall commercial development rather than any specifically health-relevant retail exposure. These interpretations are not mutually exclusive, and the present ecological design cannot adjudicate between them. Establishing whether general commercial retail volume is independently health-relevant, beyond its correlation with urbanicity and deprivation, will require longitudinal and individual-level studies.

### Strengths and Limitations

This study has several strengths. First, to our knowledge, this is among the first studies to develop a reproducible approach to measuring built-environment characteristics from digital platform data at national scale. The UBER Index was derived from programmatically accessible Google Places data and can be updated using a consistent protocol. Second, we evaluated key measurement properties of the index, including internal structure, spatial distribution, and construct validity relative to established benchmarks. Third, the analysis incorporated spatial statistical methods to account for geographic dependence in county-level outcomes. Fourth, the study included sensitivity analyses, including quantile regression and comparisons across geographic scales, to evaluate the robustness of the observed association. Finally, the analysis used a large national sample of counties, allowing assessment of geographic variation across urban and rural contexts.

Several limitations should be considered. The cross-sectional ecological design prevented the assessment of temporal relationships because exposure and outcome were measured concurrently and precluded causal or individual-level inference. As discussed above, the modifiable areal unit analysis revealed sign-discordant results across geographic scales, indicating ecological confounding. Convergent validity with established food-environment benchmarks was weak. Several sources of exposure measurement error also warrant caution: Google Places coverage depends on business registration and platform completeness, which vary geographically and may underrepresent rural areas and informal food sources; establishments may be misclassified across categories; and counts derived from centroid-based Nearby Search queries may incompletely capture large or irregularly shaped counties. Consistent with this, data were missing for a substantial share of counties, and excluded counties were markedly more rural than those retained (Multimedia Appendix 1), so the analytic sample over-represents more urban and commercially active counties and may not generalize to the most rural areas, where measurement gaps are greatest. Because missingness in Google Places coverage was likely related to county characteristics such as rurality rather than occurring completely at random, excluded counties may differ systematically from included counties. In addition, establishment counts were derived from a fixed 3-km centroid catchment rather than complete county boundary enumeration. This approach may underrepresent establishments in geographically large counties and include some establishments located just outside county boundaries, although any resulting misclassification is expected to be nondifferential with respect to diabetes prevalence. Although we assessed potential selection bias by comparing included and excluded counties on geographic and demographic characteristics, some residual bias cannot be excluded. We were also unable to adjust for individual-level determinants of diabetes such as dietary behavior, physical activity, and access to health care, which may confound area-level associations. Finally, diabetes prevalence estimates were derived from CDC PLACES small-area models based on BRFSS data, and the uncertainty of these model-based estimates was not propagated into the spatial models.

### Public Health Implications and Future Research

For public health practitioners, API-derived commercial data may provide a useful complement to traditional built-environment surveillance systems. Unlike conventional datasets that are often updated at multiyear intervals, digital platform data may support more timely monitoring of changes in local retail environments. This could help identify communities undergoing rapid environmental change and provide additional context for understanding geographic variation in chronic disease burden and health inequities. More broadly, the framework contributes to digital epidemiology and the study of commercial determinants of health by offering a structured approach to measuring retail environments across large geographic areas. However, the UBER Index should not currently be used for policy decisions or resource allocation. Rather, it should be viewed as a tool for environmental health surveillance and hypothesis generation.

Several avenues for future research remain important. First, longitudinal studies are needed to determine whether temporal changes in the UBER Index correspond to subsequent changes in diabetes prevalence and other health outcomes. Second, validation studies should compare Google Places−derived measures with ground-truth assessments and alternative commercial datasets to evaluate accuracy and temporal stability. Third, future studies should derive exposure measures directly at the census-tract or neighborhood level to evaluate whether associations observed at the county level persist across geographic scale. Finally, linking API-derived environmental measures with individual-level health data may help clarify whether the observed associations reflect true environmental effects or broader contextual characteristics associated with urbanization and socioeconomic conditions.

### Conclusions

Using programmatically accessible Google Places data, we developed the UBER Index as a scalable measure of county-level commercial retail infrastructure. This study is innovative because it shows how updated digital platform data can extend built-environment surveillance beyond static food-access measures. The index captures the broader commercial establishment volume and concentration and reveals spatial, distributional, and scale-dependent patterns relevant to diabetes research. It brings a new digital surveillance approach to built-environment epidemiology. In practice, it may help public health agencies monitor changing retail environments, while requiring longitudinal and individual-level validation before policy or practice use.

## Supplementary material

10.2196/95977 Multimedia Appendix 1 Comparison of included versus excluded counties.

10.2196/95977 Checklist 1 STROBE checklist.

## Abbreviations

ADI: Area Deprivation Index
BRFSS: Behavioral Risk Factor Surveillance System
CDC: Centers for Disease Control and Prevention
MCAR: missing completely at random
NCHS: National Center for Health Statistics
OLS: ordinary least squares
PCA: principal component analysis
SEM: spatial error model
SNAP: Supplemental Nutrition Assistance Program
STROBE: Strengthening the Reporting of Observational Studies in Epidemiology
UBER: US Built Environment Retail
USDA: United States Department of Agriculture
