# Supplementary material for: Google Places API–Based US Built Environment Retail (UBER) Index and Its Spatial Association With Diabetes Prevalence Across US Counties: Cross-Sectional Ecological Study
Source: J Med Internet Res. 2026 Oct 7;28:e95977. doi: 10.2196/95977 (PMC13645318; doi:10.2196/95977)
Supplement: Supplementary file 1 [file jmir-v28-e95977-s001.pdf]

Comparison of Included Versus Excluded Counties in a Cross-Sectional Ecological Study of the US Built Environment Retail (UBER) Index and County-Level Age-Adjusted Diabetes Prevalence, Contiguous United States, 2023–2026.

Included counties (n=1,701) had complete Google Places exposure, CDC PLACES diabetes, and covariate data; excluded counties (n=1,256) were contiguous-US counties with CDC PLACES estimates but missing one or more exposure or covariate variables. Differences in continuous measures were tested with the Welch t test and in the rurality distribution with the χ^2 test.

Characteristic	Included (n=1,701)	Excluded (n=1,256)	P value
Age-adjusted diabetes prevalence, mean % (SD)	11.0 (2.2)	11.4 (2.4)	<.001
Rural county (NCHS categories 5–6), %	45.4	85.0	<.001
Area Deprivation Index, mean percentile	64.2	50.4 (largely missing)[a]	—
Census region: Northeast, %	6.4	3.3	
Census region: Midwest, %	31.2	41.7	
Census region: South, %	45.6	42.0	
Census region: West, %	16.8	12.8	

[a]The ADI was itself missing for most excluded counties (available for 34 of 1,256), so this comparison is not interpretable and no test is reported. Abbreviations: NCHS, National Center for Health Statistics urban–rural classification. Data sources: Google Places API (2026); CDC PLACES (2025 release); Area Deprivation Index national percentile (2023).
