# Supplementary material for: Google Places API–Based US Built Environment Retail (UBER) Index and Its Spatial Association With Diabetes Prevalence Across US Counties: Cross-Sectional Ecological Study
Source: J Med Internet Res. 2026 Oct 7;28:e95977. doi: 10.2196/95977 (PMC13645318; doi:10.2196/95977)
Supplement: Supplementary file 2 [file jmir-v28-e95977-s002.docx]

**Manuscript Title:** Google Places Application Programming Interface-Based United States Built Environment Retail (UBER) Index and Its Spatial Association with Diabetes Prevalence Across United States Counties: A Cross-Sectional Ecological Study

|  | | Item No. | | Recommendation | Page  No. | | | | Relevant text from manuscript |
| --- | --- | --- | --- | --- | --- | --- | --- | --- | --- |
| **Title and abstract** | | 1 | | (*a*) Indicate the study’s design with a commonly used term in the title or the abstract | 1-3 | | | | Title states “Cross-Sectional Ecological Study.” Structured abstract (442 words) |
|  |  |  |  | (*b*) Provide in the abstract an informative and balanced summary of what was done and what was found |  |  |  |  |  |
| Introduction | | | | | | | | |  |
| Background/rationale | | 2 | | Explain the scientific background and rationale for the investigation being reported | 4-5 | | | | Introduction section - paragraphs 1-3 on built environment, commercial determinants of health, measurement gap described. |
| Objectives | | 3 | | State specific objectives, including any prespecified hypotheses | 6 | | | | Last paragraph of the introduction – described aim, four objectives and testable hypotheses. |
| Methods | | | | | | | | |  |
| Study design | | 4 | | Present key elements of study design early in the paper | 6 | | | | cross-sectional ecological study, reported. |
| Setting | | 5 | | Describe the setting, locations, and relevant dates, including periods of recruitment, exposure, follow-up, and data collection | 6 | | | | contiguous United States; Google Places API (February 2026); CDC PLACES (2025); ADI (2023). |
| Participants | | 6 | | (*Cross-sectional study*—Give the eligibility criteria, and the sources and methods of selection of participants | 7 | | | | unit of analysis = county; complete enumeration; a priori exclusions; complete-case inclusion. |
| Variables | | 7 | | Clearly define all outcomes, exposures, predictors, potential confounders, and effect modifiers. Give diagnostic criteria, if applicable | 7-8 | | | | Exposure (UBER Index), Outcome (age-adjusted diabetes prevalence), Covariates (ADI, urbanicity, census division) |
| Data sources/ measurement | | 8* | | For each variable of interest, give sources of data and details of methods of assessment (measurement). Describe comparability of assessment methods if there is more than one group | 8-10 | | | | Exposure (Google Places Nearby Search; four categories and rationale), Outcome (CDC PLACES/BRFSS), Covariates. |
| Bias | | 9 | | Describe any efforts to address potential sources of bias | 11 | | | | Added: included-vs-excluded comparison; Multimedia Appendix 1; Discussion, Limitations (coverage, misclassification, selection |
| Study size | | 10 | | Explain how the study size was arrived at | 11 | | | | Complete enumeration; final analytic sample of 1,701 of 2,957 contiguous-US counties (57.5%). |
| Quantitative variables | 11 | | Explain how quantitative variables were handled in the analyses. If applicable, describe which groupings were chosen and why | | | 11 | | winsorization, log transformation, z-standardization, and PCA; Spatial Modeling. | |
| Statistical methods | 12 | | (*a*) Describe all statistical methods, including those used to control for confounding | | | 12 | | Spatial Modeling and Sensitivity Analyses: SEM (GM-error), OLS baseline, quantile regression | |
|  |  |  | (*b*) Describe any methods used to examine subgroups and interactions | | | 12 | | tract-vs-county level analysis | |
|  |  |  | (*c*) Explain how missing data were addressed | | | 12 | | MCAR | |
|  |  |  | (*d*) *Cohort study*—If applicable, explain how loss to follow-up was addressed  *Case-control study*—If applicable, explain how matching of cases and controls was addressed  *Cross-sectional study*—If applicable, describe analytical methods taking account of sampling strategy | | | 13 | | spatial autocorrelation; spatial error model | |
|  |  |  | (*e*) Describe any sensitivity analyses | | | 12 | | Quantile regression, outcome-resolution sensitivity analysis | |
| Results | | | | | | | | | |
| Participants | 13* | | (a) Report numbers of individuals at each stage of study—eg numbers potentially eligible, examined for eligibility, confirmed eligible, included in the study, completing follow-up, and analysed | | | 14 | | Sample: 1,701 counties included, 1,256 excluded with reasons; Multimedia Appendix 1. | |
|  |  |  | (b) Give reasons for non-participation at each stage | | | - | | No human subjects included | |
|  |  |  | (c) Consider use of a flow diagram | | | - | | Provided flow chart (Figure 1) | |
| Descriptive data | 14* | | (a) Give characteristics of study participants (eg demographic, clinical, social) and information on exposures and potential confounders | | | 14 | | Study Sample and Index Measure Properties | |
|  |  |  | (b) Indicate number of participants with missing data for each variable of interest | | | 15 | | Multimedia Appendix 1 (included vs excluded). | |
|  |  |  | (c) *Cohort study*—Summarise follow-up time (eg, average and total amount) | | | - | | Not applicable | |
| Outcome data | 15* | | *Cross-sectional study—*Report numbers of outcome events or summary measures | | | 15 | | age-adjusted diabetes prevalence distribution and spatial autocorrelation (Moran's I). | |
| Main results | 16 | | (*a*) Give unadjusted estimates and, if applicable, confounder-adjusted estimates and their precision (eg, 95% confidence interval). Make clear which confounders were adjusted for and why they were included | | | 16-17 | | Spatial Error Model and Area deprivation: estimates with 95% CIs; outcome-specificity (negative-control) result. | |
| Other analyses | 17 | | Report other analyses done—eg analyses of subgroups and interactions, and sensitivity analyses | | | 18 | Quantile Regression (five quantiles with 95% CIs) and Scale Sensitivity (county vs tract). | | |
| Discussion | | | | | | | | | |
| Key results | 18 | | Summarise key results with reference to study objectives | | | 18 | First paragraph | | |
| Limitations | 19 | | Discuss limitations of the study, taking into account sources of potential bias or imprecision. Discuss both direction and magnitude of any potential bias | | | 23 | Included in strengths and limitations section | | |
| Interpretation | 20 | | Give a cautious overall interpretation of results considering objectives, limitations, multiplicity of analyses, results from similar studies, and other relevant evidence | | | 22-23 | ecological/MAUP interpretation; convergent-validity discussion; public-health implications | | |
| Generalisability | 21 | | Discuss the generalisability (external validity) of the study results | | | 22 | In the limitation section added selection toward more urban counties and also Multimedia Appendix 1 | | |
| Other information | | | | | | | | | |
| Funding | 22 | | Give the source of funding and the role of the funders for the present study and, if applicable, for the original study on which the present article is based | | | 26 | Included at the end of the manuscript | | |

*Give information separately for cases and controls in case-control studies and, if applicable, for exposed and unexposed groups in cohort and cross-sectional studies.

**Note:** An Explanation and Elaboration article discusses each checklist item and gives methodological background and published examples of transparent reporting. The STROBE checklist is best used in conjunction with this article (freely available on the Web sites of PLoS Medicine at http://www.plosmedicine.org/, Annals of Internal Medicine at http://www.annals.org/, and Epidemiology at http://www.epidem.com/). Information on the STROBE Initiative is available at www.strobe-statement.org.
